# Advances of Digital Detection for Foodborne Pathogens

**DOI:** 10.3390/foods15071250

**Published:** 2026-04-06

**Authors:** Ruonan He, Diming Hua, Wenwen Wu, Mojun Shi, Xuejiao Huang, Xuhan Xia, Ruijie Deng

**Affiliations:** 1 College of Ecology and Environment, Chengdu University of Technology, Chengdu 610059, China; 2 College of Biomass Science and Engineering, Sichuan University, Chengdu 610065, China; 3 Key Laboratory of Baijiu Supervising Technology, State Administration for Market Regulation, Chengdu 610000, China

**Keywords:** foodborne pathogen, digital detection, nucleic acid, biosensing, food safety

## Abstract

The implementation of stringent regulatory policies for foodborne pathogens necessitates ultra-sensitive analytical methods. Digital detection, characterized by absolute quantification and tolerance to complex matrices, serves as a robust approach for food safety monitoring. This review summarizes recent advances in digital detection for foodborne pathogens, including nucleic acid amplification-based platforms such as droplet digital PCR and digital isothermal amplification, as well as emerging preamplification-free approaches based on enzyme-mediated signal conversion, functional nanomaterials, and microfluidic devices. We also profile the applications of digital detection technologies for achieving highly specific and accurate detection of foodborne pathogens and discuss their capabilities in viable bacteria quantification, antimicrobial resistance analysis, and multiplex detection. We finally discuss emerging trends, including partition-free digital detection and artificial intelligence-assisted analysis. These advances are expected to promote the development of intelligent and data-driven food safety surveillance strategies.

## 1. Introduction

Foodborne diseases impose a substantial global health burden from both epidemiological and economic perspectives. According to the World Health Organization, these pathogens cause approximately 600 million illnesses and 420,000 deaths annually, underscoring their severe impact on global morbidity and mortality [1,2]. As the primary causative agents of these illnesses, their threat is further amplified by the interconnected global food network, which accelerates pathogen spread and frequently turns localized contaminations into widespread, cross-border public health emergencies [3,4,5]. Beyond their public health impact, these pathogens inflict devastating economic costs driven by massive healthcare expenses and productivity losses, alongside severe financial repercussions for the food industry due to extensive product recalls and reputational damage [6,7]. As a result, many regulatory frameworks adopt a “zero-tolerance” policy toward specific foodborne pathogens, necessitating ultra-sensitive analytical methods capable of identifying trace levels of residual pathogens within food samples to enable surveillance before outbreak occurrence [8]. Although culture-based microbiological methods provide reliable and definitive results, their inherently long turnaround times often exceed regulatory decision windows, thereby delaying timely intervention [9]. Molecular approaches offer a faster alternative but struggle with the intrinsic complexity of food matrices. Components such as lipids, polysaccharides, and the competitive microbiota found in fermented products often interfere with detection [10], leading to signal attenuation, nonspecific interference, and false negatives [11,12,13]. These limitations drive a need for robust molecular tools explicitly tailored for complex food matrices. To advance pathogen surveillance, researchers must mitigate severe matrix interference without exhaustive pretreatment and translate laboratory-grade sensitivity into rapid, field-deployable formats.

Conventional molecular strategies for pathogen detection typically encompass volatile organic compounds (VOCs) analysis, immunoassays, and nucleic acid-based detection methods [14,15,16,17]. VOCs-based detection enables rapid and noninvasive identification of foodborne pathogens by profiling metabolic signatures, but its specificity and robustness are often compromised by strain variability and interference from complex food matrices [18,19]. Immunoassays offer rapid readouts but typically suffer from limited sensitivity and cross-reactivity [20]. Nucleic acid-based methods, particularly quantitative polymerase chain reaction (qPCR) and isothermal amplification techniques, have improved analytical sensitivity and specificity, enabling rapid and sequence-specific detection of foodborne pathogens [8,21,22]. Nevertheless, nucleic acid-based assays remain challenged by amplification-associated false-positive contamination and elevated background signals, which can compromise detection accuracy and sensitivity [10,23]. In contrast, digital nucleic acid assays achieve absolute quantification by partitioning samples into thousands of independent reactions [24], thereby substantially improving quantitative accuracy and analytical sensitivity [25], while simultaneously enhancing tolerance to complex food matrices and enabling single-molecule resolution.

In this review, we comprehensively summarize recent advances in digital detection techniques for foodborne pathogen analysis. We focus on both nucleic acid amplification-enabled and preamplification-free digital tools, elucidating their fundamental principles, technological implementations, and representative applications in food samples. Particular emphasis is placed on approaches that enable absolute quantification, viability-relevant detection, and field-deployable analysis. Finally, current challenges and future opportunities for integrating digital detection with emerging microfluidic, novel enzymatic biosensors, and artificial intelligence (AI) technologies are discussed, aiming to provide a conceptual framework for next-generation food safety diagnostics.

## 2. Nucleic Acid Amplification-Based Digital Detection

Nucleic acid amplification technologies constitute a cornerstone of molecular biology by enabling the highly specific enrichment of target gene sequences, thereby enhancing analytical sensitivity [26]. When integrated with digital detection principles, these amplification strategies allow absolute quantification of target molecules through sample partitioning, effectively eliminating the reliance on quantification calibration curves and substantially improving measurement accuracy and reproducibility [27]. In this section, we review the major classes of amplification-based digital nucleic acid detection technologies, spanning from digital droplet PCR to emerging digital formats of isothermal nucleic acid amplification, and highlight their applications in the sensitive and reliable detection of foodborne pathogens.

### 2.1. Droplet Digital PCR Tools

**Figure 1:**
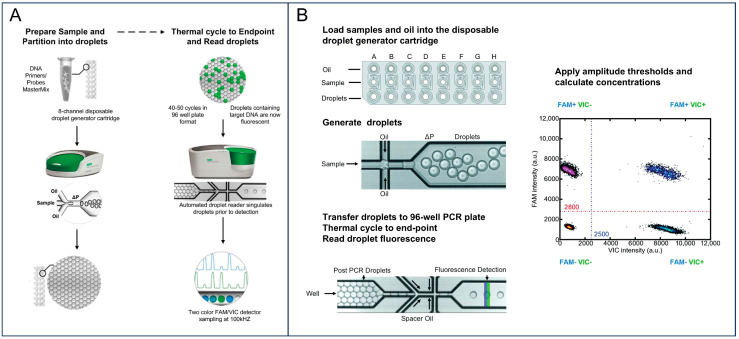
The workflow of droplet digital PCR. (**A**) The reaction mixture is partitioned into water-in-oil droplets using a microfluidic droplet generator cartridge, followed by end-point thermal cycling in a 96-well plate. After amplification, droplets are sequentially analyzed by a fluorescence droplet reader for single-droplet signal detection. Adapted with permission from Bio-Rad Laboratories, Inc. (**B**) High-throughput ddPCR simultaneously processes eight samples using an eight-channel droplet generator, followed by droplet-based amplification and fluorescence counting for Poisson-based absolute quantification. Reprinted with permission from ref. [25].

Droplet digital PCR (ddPCR), as an advanced PCR technology, represents a fundamental shift in nucleic acid detection from analog signal measurement to digital signal readout. The ddPCR enables absolute quantification of nucleic acids by discretizing a sample into a large array of isolated reaction compartments, within which target molecules are randomly distributed [28,29]. When sufficiently diluted, each compartment contains either no target or a single target molecule [30] (Figure 1A). After endpoint amplification, compartments are classified as signal-positive or -negative, and the initial target concentration is inferred from the proportion of positive reactions using Poisson-based statistical analysis. Owing to its calibration-free nature and high quantitative precision, ddPCR has emerged as a powerful tool for food safety testing, particularly for detecting low-abundance pathogens in complex food matrices.

With increasing technological maturity, ddPCR has been increasingly applied to nucleic acid detection in highly complex biological matrices [31,32,33,34]. A TaqMan chemistry assay for the ddPCR platform targeting mtDNA was developed, wherein performance for target detection and quantification was defined according to detection probability and measurement precision, respectively [35]. Based on these predefined criteria, threshold concentrations were determined to classify samples as target-positive, corresponding to the analytical limit of detection (LOD), and to identify the lowest concentration at which accurate quantification could be reliably achieved, defined as the analytical lower limit of quantification. The ddPCR has the potential to achieve highly sensitive analysis by precisely targeting and quantifying the specific DNA sequences of various foodborne pathogens. In ddPCR, amplification occurs within isolated droplets, ensuring that any amplicons detected from a droplet containing a single bacterial cell must originate from that cell. This compartmentalization enables the simultaneous assessment of multiple genetic targets at the single-cell level, allowing the determination of whether different genes coexist within the same genome. Such capability is particularly important for defining the virotype of *E. coli*, which depends on the co-occurrence of specific virulence determinants. By directly introducing intact bacterial cells rather than extracted DNA into the reaction system, ddPCR has been employed to detect multiple virulence factors within individual *E. coli* cells [36]. Furthermore, ddPCR has played an important role in the surveillance of antimicrobial-resistant strains within food systems. The robustness of endpoint detection against sample-derived interference enables accurate quantification of low-abundance resistance genes in complex microbial backgrounds. For example, the technology has been successfully applied to the precise absolute quantification of critical resistance determinants such as carbapenemase genes in retail meat and aquatic products [37]. Moreover, the partitioning mechanism of ddPCR facilitates multiplexed genetic analysis [25,38]. By assessing the co-occurrence of resistance genes and species-specific markers within the same droplets, researchers can distinguish between resistance harbored by viable pathogens and that arising from free environmental DNA, thereby refining risk assessments for antimicrobial resistance transmission.

To meet the high-throughput demands of modern food safety surveillance, increasing attention has been directed toward overcoming the optical channel limitations inherent to conventional ddPCR platforms. By integrating multiplex detection across multiple fluorescence channels, ddPCR platforms can substantially improve target throughput and analytical capacity (Figure 1B). One effective strategy to achieve higher-order multiplexing involves modulating fluorescence signal amplitudes by titrating the concentrations of target-specific probes. This approach generates stratified fluorescence clusters within a single detection channel, thereby producing distinct signal “fingerprints” for individual targets. Such intensity-based multiplexing enables the simultaneous detection of multiple major foodborne pathogens, including *Salmonella* spp., *Listeria monocytogenes* (*L. monocytogenes*), and *Staphylococcus aureus* (*S. aureus*), or the parallel profiling of multiple serotype-specific markers within a single reaction well [25,39]. Incorporation of multiplexing strategies into the ddPCR framework not only improves sample utilization and reduces operational costs but also provides enhanced resolution for characterizing polymicrobial contamination, addressing a key analytical bottleneck in complex food processing environments.

Although ddPCR offers highly accurate, absolute quantification and robust tolerance to PCR inhibitors in complex food matrices, practical barriers limit its routine industrial application [40,41]. Primary drawbacks include high instrumentation and consumable costs, alongside a multi-step workflow that requires trained personnel. Primary drawbacks include high instrumentation and consumable costs, alongside a multi-step workflow that requires trained personnel. Consequently, ddPCR is largely unsuitable for on-site analysis. Furthermore, its moderate throughput and extended turnaround times are often insufficient to meet the rapid screening demands of high-volume food processing environments.

### 2.2. Digital Isothermal Amplification Detection

Digital isothermal amplification has emerged as a compelling alternative to conventional ddPCR. By eliminating the need for precise thermal cycling, isothermal amplification relies on constant-temperature amplification chemistries, such as loop-mediated isothermal amplification (LAMP) [42], recombinase polymerase amplification (RPA) [43], and rolling circle amplification (RCA) [44], that are compartmentalized into discrete microreactors for digital readout. This approach substantially simplifies system hardware while markedly reducing assay turnaround time, thereby enhancing its suitability for point-of-need testing and food safety surveillance. Recent developments in digital isothermal amplification have focused on addressing the intrinsic limitations of conventional digital assays through innovations in reaction compartmentalization, material design, and integration with sequence-specific recognition elements [45,46,47], resulting in improved robustness, sensitivity, and functional adaptability.

**Figure 2:**
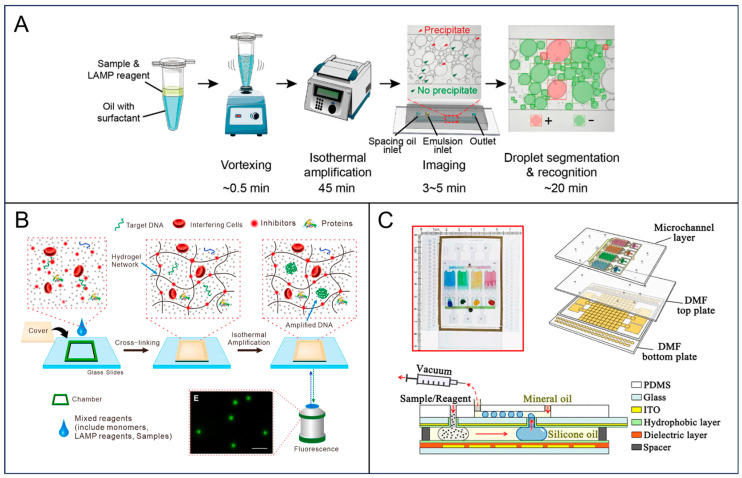
Isothermal amplification for digital detection. (**A**) Schematic of digital LAMP, where vortex-generated emulsions undergo isothermal amplification and flow-cell imaging, followed by deep learning-based occupancy analysis for absolute nucleic acid quantification, with spacing oil preventing droplet packing. Reprinted with permission from ref. [48]. (**B**) Schematic of isothermal amplification inside the nanoporous hydrogel for digital LAMP in complex matrices. Reprinted with permission from ref. [49]. (**C**) Schematic of the LAMP-integrated digital-to-droplet microfluidic system and its operational workflow for digital nucleic acid detection. Reprinted with permission from ref. [50].

One of the primary barriers to the widespread adoption of digital assays is the stringent requirement for monodisperse droplet generation, which necessitates complex microfluidic chips and flow control systems. To circumvent this, Chen et al. proposed a “Deep-dLAMP” strategy that fundamentally relaxes the hardware constraints [48]. Instead of relying on uniform droplets, they utilized a simple vortex-generated polydisperse emulsion system. By training a deep learning algorithm (Mask R-CNN) to recognize and analyze droplets of varying sizes, they successfully achieved absolute quantification based on Poisson statistics despite the volume heterogeneity. This approach demonstrated that software intelligence can compensate for hardware simplicity, enabling accurate nucleic acid quantification with a LOD as low as 5.6 copies/µL without expensive droplet generators (Figure 2A). Beyond instrumentation, sample preprocessing remains a bottleneck in digital detection techniques, especially for food samples rich in polymerase inhibitors. Addressing this challenge, Yi et al. developed a nanoporous hydrogel-based digital LAMP system capable of direct analysis in untreated complex matrices [49]. The key innovation is the hydrogel’s size-exclusion property, which serves as a self-cleaning filter that allows reagents and target nucleic acids to diffuse while blocking larger inhibitory species typically found in complex matrices such as whole blood and milk-containing samples (Figure 2B). This “sample-in-answer-out” capability in a digital format significantly streamlines the workflow for foodborne pathogen detection in realistic environments. This method can detect *E. coli*, *Salmonella typhimurium* (*S. typhimurium*), and *Listeria monocytogenes* (*L. monocytogenes*) in complex samples within 20 min, making it a promising tool for sensitive and on-site food sampling inspections. To further enhance throughput and automation, the convergence of digital microfluidics with droplet microfluidics has led to integrated “Digital-to-Droplet” platforms. Xie et al. introduced a hybrid device designed to automate the entire pipeline from nucleic acid extraction to digital quantification [50]. Subsequent to sample loading, the DMF module autonomously executes the isolation of nucleic acids and manages the precise metering of the eluate for integration with various amplification cocktails (Figure 2C). These prepared reaction mixtures are then seamlessly transferred to the droplet generation module for massive parallel amplification, enabling the multiplexed detection of pathogens. This fully automated “lab-on-a-chip” workflow effectively circumvents the variability and contamination risks associated with fragmented manual protocols.

**Figure 3:**
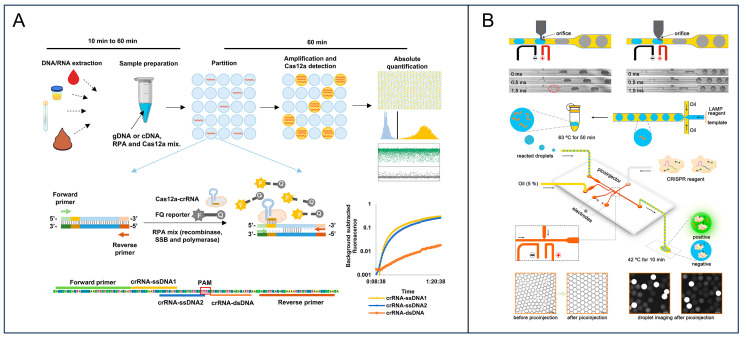
CRISPR-based isothermal amplification for digital detection. (**A**) Schematic of the CRISPR-based approach for digital quantification of nucleic acid. In each partition, the DNA is amplified by RPA and detected by Cas12a-crRNA, resulting in a fluorescent signal in the partition. Following endpoint fluorescence detection, the fraction of positive partitions is quantified, and target concentrations are estimated according to Poisson statistics. Reprinted with permission from ref. [46]. (**B**) Microfluidic picoinjection-assisted DropCRISPR platform, including picoinjector structures, droplet injection dynamics, hybrid LAMP–CRISPR/Cas12a workflow, and representative brightfield and fluorescence images before and after signal generation. Reprinted with permission from ref. [54].

While traditional isothermal techniques, such as LAMP and RPA, offer rapid amplification kinetics, their digital implementation is frequently impeded by non-specific background signals arising from primer dimers and spurious off-target amplification [51]. The integration of clustered regularly interspaced short palindromic repeats (CRISPR)-associated (Cas) systems has revolutionized digital isothermal amplification by adding a procedure of sequence-specific signal recognition [52]. In this strategy, Cas effectors such as Cas12 and Cas13 are guided by target-specific CRISPR RNAs, which require sequence complementarity to activate their collateral *trans*-cleavage activity. Upon activation, the Cas effectors cleave surrounding reporter probes, thereby achieving highly specific signal amplification [53]. A digital CRISPR-based method that combines RPA with Cas12a collateral cleavage was developed [46]. The preamplification by RPA triggers the Cas12a *trans*-cleavage activity upon target recognition, generating a fluorescent signal that is strictly dependent on the specific target sequence, thereby achieving absolute quantification with high specificity (Figure 3A). Similarly, Wu et al. developed “DropCRISPR”, a two-step microfluidic system pairing LAMP with Cas12a [54]. This platform separates the amplification and detection phases to optimize the conditions for both enzymes, thereby enabling the ultrasensitive detection of *S. typhimurium*. Specifically, the assay achieved a detection limit at the fM level for the *invA* gene and down to 10^2^ CFU/mL in bacterial culture (Figure 3B). They also demonstrate that the DropCRISPR assay can analyze *S. typhimurium* in raw milk samples without additional nucleic acid extraction. The digital partitioning of the CRISPR reaction effectively confines the background noise, significantly enhancing the signal-to-noise ratio compared to bulk assays.

**Figure 4:**
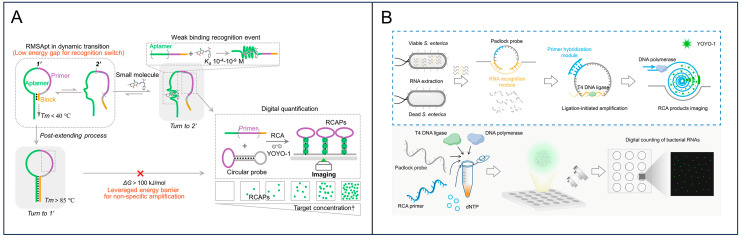
RCA-based partition-free digital detection. (**A**) Schematic of the RMSApt and its application for digitally quantifying small molecules. Reprinted with permission from ref. [58]. (**B**) Schematic of the digital RCA assay for detecting viable foodborne pathogens. Reprinted with permission from ref. [59].

Unlike digital LAMP or RPA assays that necessitate the physical compartmentalization of bulk samples into discrete micro-reactors, RCA inherently generates localized DNA nanoflowers in a “one-target-one-amplicon” manner, thereby enabling partition-free digital quantification [55,56,57]. Our group developed a digital RCA strategy coupled with aptamer-based recognition, which transduces small molecule recognition into quantifiable DNA signals [58]. In this system, target-induced conformational changes in the aptamer release a primer to trigger digital amplification, enabling the absolute quantification of food contaminants (Figure 4A). We also advanced the field by developing a digital RCA (dRCA) assay targeting bacterial RNA [59]. By using a ligation-dependent padlock probe that only circularizes upon perfect hybridization with the target RNA, which degrades rapidly in dead cells, the dRCA specifically quantifies viable bacteria. The dRCA enables high sensitivity of 10 CFU/mL and maintains a wide quantitative dynamic range of 6 orders of magnitude. This assay can detect viable *Salmonella* at proportions as low as 0.1%, exhibiting approximately 50-fold higher sensitivity than conventional live/dead staining methods (Figure 4B). The method was successfully applied for the sensitive detection of viable bacteria in pasteurized milk, demonstrating its potential as a new tool for evaluating pasteurization efficiency in the food industry.

By eliminating the requirement for complex thermal cycling, digital isothermal amplification provides a cost-efficient and field-deployable alternative to ddPCR. These assays enable rapid turnaround times, facilitating point-of-need food safety surveillance. Nevertheless, a persistent challenge is their susceptibility to non-specific background amplification, an artifact often exacerbated within digital partitions [60,61]. Although integrating CRISPR/Cas effectors effectively circumvents this specificity bottleneck, it introduces higher reagent costs and methodological complexity. While the intricate design of isothermal primers may currently restrict widespread adoption, ongoing advancements in enzyme engineering and assay integration firmly position this technology as a highly promising foundation for rapid and sensitive pathogen monitoring.

## 3. Preamplification-Free Digital Analysis

Preamplification-free digital detection techniques circumvent the critical bottlenecks of traditional nucleic acid amplification, such as amplification bias, aerosol cross-contamination, and false-positive results, by implementing direct counting of target molecules [62]. This approach substantially simplifies analytical workflows and reduces dependence on complex thermal cycling instrumentation, while preserving high quantitative accuracy and single-molecule-level resolution by relying on signal transduction rather than target replication. Accordingly, this section summarizes recent advances in preamplification-free digital detection strategies, which are broadly categorized into enzyme-mediated signal conversion, functional nanomaterial-assisted sensing, and microfluidic architecture-based platforms.

### 3.1. Enzyme-Mediated Signal Transduction Systems

Harnessing the intrinsic catalytic capabilities of enzymatic proteins offers an effective approach for achieving signal amplification without the prerequisite of nucleic acid replication [63,64,65]. Unlike polymerase-based methods that depend on exponential copying of target sequences and are therefore prone to error accumulation and contamination, protein-mediated strategies separate target recognition from signal generation. By utilizing the target nucleic acid as a guide to trigger the enzymatic processing of abundant reporter substrates, these systems can generate intense and quantifiable signals from single molecular events.

**Figure 5:**
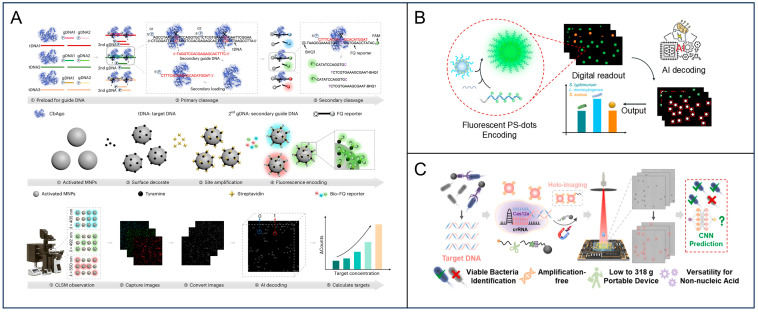
Preamplification-free digital detection based on enzyme- and advanced material-mediated signal enhancement. (**A**) Schematic of the analytical platform integrating CbAgo-mediated two-step cleavage, magnetic bead-based fluorescence encoding, and AI-assisted image decoding for target quantification. Reprinted with permission from ref. [66]. (**B**) A preamplification-free digital sensing strategy utilizing botryoidal-like fluorescent polystyrene dots (PS-dots) for the multiplexed identification of pathogenic bacteria. Reprinted with permission from ref. [67]. (**C**) Schematic of a holography-integrated biosensing platform for preamplification-free nucleic acid detection, featuring high-accuracy and fast deep-learning-assisted YOLOv7 object detection for PS microsphere signal readout. Reprinted with permission from ref. [68].

This strategy is exemplified by the recent integration of Argonaute proteins into digital biosensing platforms. Wang et al. engineered a digital carrier system, termed d-MAGIC, which leverages the programmable nuclease activity of mesophilic *Clostridium butyricum* Argonaute (CbAgo) for the multiplexed detection of foodborne pathogens [66]. In this assay, the specific hybridization of genomic DNA targets guides CbAgo to execute the precise, continuous cleavage of fluorescence-quencher reporters immobilized on magnetic beads (Figure 5A). Unlike conventional digital methods that rely on individual droplets and microfluidic architectures, this study employs uniform magnetic beads as digital carriers instead of droplets. Using magnetic beads as signal-bearing units effectively minimizes background interference and avoids the drawbacks associated with water-in-oil systems, which are highly sensitive to environmental perturbation. This process effectively “transcodes” the presence of trace pathogen DNA into distinct, high-intensity fluorescent bead clusters. By circumventing the need for upstream DNA amplification, this enzyme-driven signal transduction mechanism achieved a limit of detection as low as 6 CFU/mL, ensuring exceptional quantitative fidelity even in complex matrices. By integrating programmable CbAgo with magnetic beads and AI decoding, this technique enables digital, preamplification-free, and ultrasensitive simultaneous detection of three foodborne pathogens. The practical applicability of d-MAGIC was validated using 100 diverse retail samples, including shrimp, eggs, and chicken, demonstrating high diagnostic consistency with qPCR. This tool successfully identified 16 samples positive for *S. Typhimurium*, 9 for *S. aureus*, and 12 for *L. monocytogenes*, confirming its robustness for real-world food safety monitoring.

Enzyme-mediated signal amplification, particularly through emerging cascade strategies like cascaded CRISPR systems, provides an effective alternative to traditional target replication. These preamplification-free techniques bypass limitations such as aerosol cross-contamination and amplification bias. Capable of driving detection limits down to femtomolar levels, such programmable effectors offer the broad dynamic range essential for robust, on-site pathogen surveillance. However, translating these analytical capabilities into routine practice also remains challenging. Widespread implementation is currently restricted by the high acquisition costs of specialized proteins and the intricate biochemical optimization required to sustain their catalytic stability within complex food matrices.

### 3.2. Advanced Nanomaterials for Signal Enhancement

The integration of functional nanomaterials into digital detection has emerged as an important strategy to enhance signal output and overcome the limitations of nucleic acid amplification. Owing to their tunable spectral diversity, fluorescent nanospheres have emerged as versatile signal reporters for high-order multiplexing in digital diagnostics. Notably, emissive materials such as quantum dots and upconversion nanoparticles are extensively employed as encoded probes, leveraging their distinct emission signatures to facilitate simultaneous multi-target quantification. Wang et al. pioneered a “botryoidal-like” fluorescent polystyrene dot (PS-dot) system, synthesized via a primer exchange reaction that drives the self-assembly of DNA concatemers onto polystyrene nanospheres [67]. In this assay, the target DNA fragment derived from Ago-mediated cleavage functions as an intermediary linker that physically anchors these high-intensity PS-dots to magnetic beads (Figure 5B). This “sandwich” hybridization converts a single target recognition event into the capture of a massive fluorescent cluster, thereby generating a distinct optical signature that can be digitally counted and decoded for multiplexed pathogen identification based on the specific color-size combinations of the bead-particle complexes.

The lens-free holography microscope can also be integrated with deep learning algorithms to enhance the efficiency and accuracy of the PS counting [68]. By eliminating bulky optical lenses, the lens-free holography microscope records the interference patterns of shadows cast by microscopic targets directly onto a complementary metal oxide semiconductor sensor, significantly expanding the field of view compared to traditional microscopy (Figure 5C). To resolve the complex diffraction patterns of dense samples, a deep-learning model such as a YOLO-based architecture is employed to reconstruct and count individual targets with high fidelity. YOLO, which stands for ‘You Only Look Once,’ is a highly efficient, state-of-the-art convolutional neural network model designed for real-time object detection [69,70]. The combination of nanomaterial-enhanced signal brightness with AI-assisted holographic reconstruction establishes a reliable framework for high-throughput and direct-counting detection suitable for point-of-need applications in food safety.

Highly emissive nanomaterials, such as fluorescent polystyrene or quantum dots, efficiently amplify single molecular recognition events into quantifiable optical clusters. While these techniques enable multiplexing without thermal cycling, significant manufacturing hurdles remain, particularly regarding synthesis, precise surface functionalization, and batch-to-batch consistency. Moreover, the reliance on capital-intensive imaging infrastructure and complex deep-learning algorithms for multiplexed signal decoding restricts their practical deployment in resource-constrained settings.

### 3.3. Microfluidic-Enabled Digital Analysis Systems

The structural advancement of microfluidic platforms enables preamplification-free digital quantification by physically confining analytes into micro reaction compartments. Such miniaturization markedly increases the local concentration of signal reporters, thereby improving the signal-to-noise ratio and enabling sensitive detection without enzymatic amplification.

**Figure 6:**
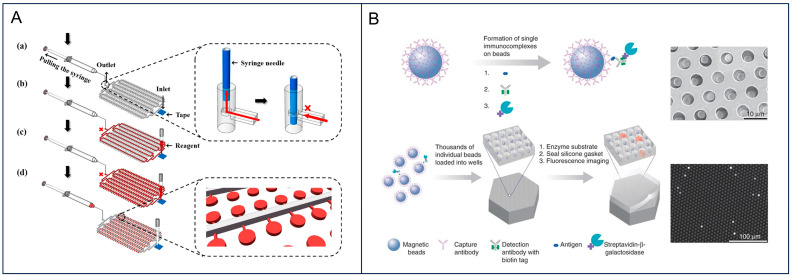
Microfluidic-enabled preamplification-free digital detection. (**A**) Schematic illustrating the operating principle of the negative pressure-driven digital microfluidic chip, utilizing a vacuum syringe for sequential solution dispersion, microwell filling, and excess fluid removal to achieve precise reagent compartmentalization. Reprinted with permission from ref. [71]. (**B**) Schematic and imaging of a digital ELISA platform, where single proteins are captured on beads and isolated in femtoliter wells to enable absolute quantification via active bead counting. Reprinted with permission from ref. [72].

A detection tool termed the “digital dipstick” was successfully developed, which streamlines the digital detection workflow into a “sample addition-incubation-counting” process and enables direct on-site test of pathogenic bacteria. The “digital dipstick” is a spoon-shaped plastic device fabricated from polymethyl methacrylate sheets with a thickness of approximately 1 mm. By using miniaturized hydrophobic-hydrophilic partitions, the device autonomously captures individual *E. coli* for in situ growth, effectively bypassing the need for complex off-chip pre-concentration. This concept was recently elevated by Quan et al., who integrated a digital microfluidic platform with a Time-Lapse images driven EfficientNet-Transformer Network (TLENTNet) [71]. The deep learning framework extracts high-dimensional spatiotemporal features from the growing colonies, effectively creating a unique “phenotypic fingerprint” for each bacterial species. By analyzing subtle inter-species variations in colony expansion trajectories, edge roughness, and optical density fluctuations, this method can accurately discriminate between co-cultured pathogens such as *Salmonella* and *E. coli* O157:H7 without biochemical labeling (Figure 6A). In addition, this AI-empowered platform achieved a classification accuracy exceeding 96% and a LOD down to 1 CFU/mL, demonstrating that computational intelligence can effectively compensate for the absence of biochemical preamplification in multiplexed diagnostics.

The versatility of microfluidic compartmentalization extends beyond bacterial digital culture, enabling the preamplification-free digital quantification of diverse biomolecules ranging from bacterial proteins to nucleic acids. For instance, Digital enzyme-linked immunosorbent assay platforms leverage femtoliter-sized microwell arrays to confine single enzyme-labeled immunocomplexes, creating a high local concentration of fluorescent products that facilitates single-molecule protein counting [72] (Figure 6B). The integration of CRISPR/Cas systems with microfluidic architectures enables the construction of amplification-free digital diagnostic tools. By synergizing CRISPR-Cas13-mediated molecular recognition with microchamber-array partitioning, the SATORI platform enables the digital quantification of RNA targets without the need for nucleic acid preamplification. This approach achieves an impressive analytical sensitivity, reaching a detection limit of 10 fM in just 5 min. This method can be implemented using a compact fluorescence microscope, demonstrating significant potential for widespread application in on-site testing within the food industry.

Microfluidic partitioning enables direct target counting by confining analytes into femtoliter compartments to maximize signal-to-noise ratios. Although miniaturized platform tools show strong potential for on-site pathogen screening, their transition to commercial use is hindered by complex micro-fabrication and fluid control requirements. Furthermore, advanced AI algorithms are expected to further optimize the design strategies and signal readout methods of microfluidic-based techniques.

## 4. Opportunities and Trends Driving Advances in Digital Detection

This section explores the transition of foodborne pathogen monitoring from labor-intensive laboratory assays toward intelligent, data-driven surveillance. We specifically highlight how partition-free strategies, AI-driven signal analysis, and portable preamplification-free devices will streamline high-throughput quantification and accelerate on-site detection within complex supply chains.

### 4.1. Partition-Free Digital Detection

Physical partitioning underpins digital nucleic acid detection by isolating individual target molecules for absolute quantification, yet its success strictly relies on generating highly uniform microreactors. The existing microfluidic technologies struggle to precisely control the spatiotemporal sequence of multi-step biochemical reactions. For instance, the direct co-incubation of lysis reagents with sensitive CRISPR effectors or antibodies frequently triggers premature protein degradation. While sequential reagent loading can prevent such inactivation, this approach necessitates cumbersome external micropumps and microvalves [27,73]. Relying on these complex fluidic peripherals inevitably compromises the precision of droplet manipulation, thereby severely degrading the overall reliability and analytical accuracy of the assay.

Among existing approaches, RCA can be employed to achieve this goal, enabling digital detection with one-to-one correspondence between signals and targets. The feasibility of this spatially localized amplification strategy was initially established in the field of single-cell in situ imaging. For example, our group developed a toehold-initiated RCA enabling the digital counting of individual miRNA and mRNA molecules within the cytoplasm [74]. Building on these cellular imaging successes, researchers have successfully translated this technique to food safety detection. Notable applications include the development of recognition-enhanced metastably shielded aptamers for digital quantification of small molecules [58], as well as the more recent digital RCA assay targeting 16S rRNA [59]. Looking forward, expanding partition-free digital detection strategies remains crucial for improving analytical flexibility and practical deployment. Most current systems still depend on nucleic acid amplification to achieve localized signal accumulation, which may increase assay complexity and introduce amplification-associated bias. Therefore, developing preamplification-free partition-free digital detection techniques represents an important future direction. Potential solutions include nanomaterial-assisted signal enhancement and enzyme cascade systems that transduce molecular recognition events into digital readouts. Such strategies may simplify workflows, improve robustness in complex matrices, and broaden target accessibility, thereby facilitating scalable digital detection for food safety monitoring.

### 4.2. AI-Driven Assay Design and Signal Decoding

As digital detection technologies continue to evolve toward high-throughput analysis, the complexity of experimental design and data analysis has increased substantially. Conventional threshold-based analytical approaches often struggle to address challenges such as heterogeneous background noise, signal overlap, and uneven illumination frequently encountered in complex food matrices, thereby limiting analytical accuracy and reproducibility. To overcome these optical bottlenecks, researchers are increasingly adopting advanced AI architectures for spatial signal deconvolution and adaptive noise suppression [75]. For instance, convolutional neural networks such as U-Net and Mask R-CNN have been widely implemented for the precise segmentation of dense and adjacent micro-droplets [76]. Similarly, localization algorithms such as DeepSTORM and YOLO architectures demonstrate strong capability in resolving sub-diffraction fluorescent spots and identifying overlapping signal clusters in real time [77,78]. By autonomously extracting morphological and intensity features, these computational models recover quantitative accuracy without the need for physical sample dilution. In addition, AI can also assist in the design and optimization of detection methods. Deep learning-based sequence models and statistical learning approaches are increasingly applied for target gene selection, thermodynamic optimization of degenerate primers, and prediction of off-target and cross-reactivity of CRISPR guide RNAs [79]. The convergence of AI-driven assay design with digital detection platforms is expected to facilitate the development of intelligent, robust, and scalable detection tools for foodborne pathogen monitoring.

## 5. Conclusions and Outlook

The evolution of foodborne pathogen surveillance from culture-based phenotypic identification to molecular-level digital quantification represents an important advancement for safeguarding global food safety. In this review, we systematically examined the transition from conventional amplification-based platforms, such as ddPCR and digital isothermal amplification, to emerging preamplification-free strategies driven by enzyme cascades, advanced nanomaterials, and microfluidics. These digital detection tools have demonstrated exceptional capability in achieving absolute quantification, enabling the precise characterization of pathogen viability, antimicrobial resistance, and virulence potential. To summarize these findings, Table 1 provides a comprehensive comparative analysis outlining the analytical performance, primary advantages, and practical limitations of each discussed methodology.

Despite these significant analytical achievements, several limitations currently impede the widespread industrial adoption of these techniques. While ddPCR remains the gold standard for accuracy, it is constrained by high instrumentation costs and labor-intensive, lab-dependent workflows. Conversely, although digital isothermal and preamplification-free assays simplify thermal requirements and accelerate turnaround times, they also face distinct hurdles, such as non-specific background amplification, complex novel protein design, and stability issues. Most importantly, many of these emerging methods remain confined to the laboratory testing stage, necessitating further rigorous validation and broader application across diverse, real-world food matrices to prove their reliability against complex industrial interferents.

To overcome these practical bottlenecks, future research must focus on streamlining assay architectures and enhancing in-field robustness. The recent shift toward partition-free digital quantification exemplifies this trend, effectively bypassing the hardware complexities of droplet generation. Furthermore, the integration of miniaturized imaging devices with advanced, AI-assisted analytical algorithms will be crucial for automating signal decoding and reducing the reliance on trained personnel. Ultimately, the convergence of these intelligent, portable digital sensors with ongoing innovations in protein engineering and functional materials will drive the transition from reactive contamination control toward proactive, data-driven risk management across global food supply chains.

## Figures and Tables

**Table 1:** The comparison of cascaded CRISPR-based techniques.

Assay	Analytical Technique	Target Molecules	Sensitivity	Detection Time	Refs.
Nucleic acid-based digital assay	Droplet Digital PCR (ddPCR)	DNA, RNA (via RT-ddPCR)	Single-copy level	45–90 min	[27,28,29,30,31,32,33]
	Digital LAMP	DNA	5.6 copies/µL, single bacterial level	20–45 min	[40,41]
	CRISPR-assisted isothermal amplification	DNA, RNA	0.897 copies/µL, 10^2^ CFU/mL	60–90 min	[46,47]
	Digital RCA	RNA	10 CFU/mL	30 min	[48,49]
Preamplification-free-based digital assay	Enzyme- mediated cascades	DNA, RNA	Dui, na6 CFU/mL	60 min	[54]
	Nanomaterials-assisted amplification	DNA, RNA, proteins	2–100 CFU/mL	90 min	[55,56]
	Microfluidic- enabled analysis	DNA, RNA, Proteins	14 fg/mL, 63 CFU/mL	6–7 h	[57,58]

## Data Availability

The original contributions presented in this study are included in thearticle. Further inquiries can be directed to the corresponding authors.

## References

[1] Scharff R.L. (2012). Economic burden from health losses due to foodborne illness in the United States. J. Food Prot..

[2] Springmann M., Kennard H., Dalin C., Freund F. (2023). International food trade contributes to dietary risks and mortality at global, regional and national levels. Nat. Food.

[3] Havelaar A.H., Kirk M.D., Torgerson P.R., Gibb H.J., Hald T., Lake R.J., Praet N., Bellinger D.C., de Silva N.R., Gargouri N. (2015). World Health Organization global estimates and regional comparisons of the burden of foodborne disease in 2010. PLoS Med..

[4] Pires S.M., Desta B.N., Mughini-Gras L., Mmbaga B.T., Fayemi O.E., Salvador E.M., Gobena T., Majowicz S.E., Hald T., Hoejskov P.S. (2021). Burden of foodborne diseases: Think global, act local. Curr. Opin. Food Sci..

[5] Scallan E., Hoekstra R.M., Angulo F.J., Tauxe R.V., Widdowson M.-A., Roy S.L., Jones J.L., Griffin P.M. (2011). Foodborne illness acquired in the United States—Major pathogens. Emerg. Infect. Dis..

[6] Hussain M.A., Dawson C.O. (2013). Economic impact of food safety outbreaks on food businesses. Foods.

[7] Walls H.L., Cornelsen L., Lock K., Smith R.D. (2016). How much priority is given to nutrition and health in the EU Common Agricultural Policy?. Food Policy.

[8] Law J.W.-F., Ab Mutalib N.-S., Chan K.-G., Lee L.-H. (2015). Rapid methods for the detection of foodborne bacterial pathogens: Principles, applications, advantages and limitations. Front. Microbiol..

[9] Velusamy V., Arshak K., Korostynska O., Oliwa K., Adley C. (2010). An overview of foodborne pathogen detection: In the perspective of biosensors. Biotechnol. Adv..

[10] Schrader C., Schielke A., Ellerbroek L., Johne R. (2012). PCR inhibitors–occurrence, properties and removal. J. Appl. Microbiol..

[11] Sidstedt M., Hedman J., Romsos E.L., Waitara L., Wadsö L., Steffen C.R., Vallone P.M., Rådström P. (2018). Inhibition mechanisms of hemoglobin, immunoglobulin G, and whole blood in digital and real-time PCR. Anal. Bioanal. Chem..

[12] Wu Z., Hao Z., Chai Y., Li A., Wang C., Zhang X., Chen H., Lu C. (2023). Near-infrared-excitable acetylcholinesterase-activated fluorescent probe for sensitive and anti-interference detection of pesticides in colored food. Biosens. Bioelectron..

[13] Wang Y., Salazar J.K. (2016). Culture-independent rapid detection methods for bacterial pathogens and toxins in food matrices. Compr. Rev. Food Sci. Food Saf..

[14] Liu Z., Wang M., Wu M., Li X., Liu H., Niu N., Li S., Chen L. (2023). Volatile organic compounds (VOCs) from plants: From release to detection. Trend. Anal. Chem..

[15] Gong X., Huang J., Xu Y., Li Z., Li L., Li D., Belwal T., Jeandet P., Luo Z., Xu Y. (2023). Deterioration of plant volatile organic compounds in food: Consequence, mechanism, detection, and control. Trends Food Sci. Tech..

[16] Liu S., Liao Y., Shu R., Sun J., Zhang D., Zhang W., Wang J. (2024). Evaluation of the multidimensional enhanced lateral flow immunoassay in point-of-care nanosensors. ACS Nano.

[17] Deng R., Xu L., Zhang Y., Zhang X., Yuan Z., Chen J., Xia X. (2024). CRISPR-based nucleic acid assays for food authentication. Trends Food Sci. Tech..

[18] Khatib M., Haick H. (2022). Sensors for Volatile Organic Compounds. ACS Nano.

[19] Bunge M., Araghipour N., Mikoviny T., Dunkl J., Schnitzhofer R., Hansel A., Schinner F., Wisthaler A., Margesin R., Märk Tilmann D. (2008). On-line monitoring of microbial volatile metabolites by proton transfer reaction-mass spectrometry. Appl. Environ. Microbiol..

[20] Lazcka O., Campo F.J.D., Muñoz F.X. (2007). Pathogen detection: A perspective of traditional methods and biosensors. Biosens. Bioelectron..

[21] Vinayaka A.C., Ngo T.A., Kant K., Engelsmann P., Dave V.P., Shahbazi M.-A., Wolff A., Bang D.D. (2019). Rapid detection of Salmonella enterica in food samples by a novel approach with combination of sample concentration and direct PCR. Biosens. Bioelectron..

[22] Baker Y.R., Yuan L., Chen J., Belle R., Carlisle R., El-Sagheer A.H., Brown T. (2021). Expanding the chemical functionality of DNA nanomaterials generated by rolling circle amplification. Nucleic Acids Res..

[23] Qiao J., Zhao Z., Li Y., Lu M., Man S., Ye S., Zhang Q., Ma L. (2024). Recent advances of food safety detection by nucleic acid isothermal amplification integrated with CRISPR/Cas. Crit. Rev. Food Sci..

[24] Vogelstein B., Kinzler K.W. (1999). Digital PCR. Proc. Natl. Acad. Sci. USA.

[25] Hindson B.J., Ness K.D., Masquelier D.A., Belgrader P., Heredia N.J., Makarewicz A.J., Bright I.J., Lucero M.Y., Hiddessen A.L., Legler T.C. (2011). High-throughput droplet digital PCR system for absolute quantitation of DNA copy number. Anal. Chem..

[26] Xia X., Yang H., Cao J., Zhang J., He Q., Deng R. (2022). Isothermal nucleic acid amplification for food safety analysis. Trend. Anal. Chem..

[27] Quan P.-L., Sauzade M., Brouzes E. (2018). dPCR: A technology review. Sensors.

[28] Shen F., Du W., Kreutz J.E., Fok A., Ismagilov R.F. (2010). Digital PCR on a SlipChip. Lab Chip.

[29] Salipante S.J., Jerome K.R. (2020). Digital PCR—An emerging technology with broad applications in microbiology. Clin. Chem..

[30] Pinheiro L.B., Coleman V.A., Hindson C.M., Herrmann J., Hindson B.J., Bhat S., Emslie K.R. (2012). Evaluation of a Droplet Digital Polymerase Chain Reaction Format for DNA Copy Number Quantification. Anal. Chem..

[31] Milosevic D., Mills J.R., Campion M.B., Vidal-Folch N., Voss J.S., Halling K.C., Highsmith W.E., Liu M.C., Kipp B.R., Grebe S.K.G. (2018). Applying standard clinical chemistry assay validation to droplet digital PCR quantitative liquid biopsy testing. Clin. Chem..

[32] Bogožalec Košir A., Demšar T., Štebih D., Žel J., Milavec M. (2019). Digital PCR as an effective tool for GMO quantification in complex matrices. Food Chem..

[33] Kokkoris V., Vukicevich E., Richards A., Thomsen C., Hart M.M. (2021). Challenges using droplet digital PCR for environmental samples. Appl. Microbiol..

[34] Olmedillas-López S., Olivera-Salazar R., García-Arranz M., García-Olmo D. (2022). Current and Emerging Applications of Droplet Digital PCR in Oncology: An Updated Review. Mol. Diagn. Ther..

[35] Zhu K., Suttner B., Pickering A., Konstantinidis K.T., Brown J. (2020). A novel droplet digital PCR human mtDNA assay for fecal source tracking. Water Res..

[36] He L., Simpson D.J., Gänzle M.G. (2020). Detection of enterohaemorrhagic Escherichia coli in food by droplet digital PCR to detect simultaneous virulence factors in a single genome. Food Microbiol..

[37] Carelli M., Griggio F., Mingoia M., Garofalo C., Milanović V., Pozzato N., Leoni F., Veschetti L., Malerba G., Sandri A. (2022). Detecting carbapenemases in animal and food samples by droplet digital PCR. Antibiotics.

[38] Whale A.S., Huggett J.F., Cowen S., Speirs V., Shaw J., Ellison S., Foy C.A., Scott D.J. (2012). Comparison of microfluidic digital PCR and conventional quantitative PCR for measuring copy number variation. Nucleic Acids Res..

[39] Fang Z., Zhou X., Wang X., Shi X. (2023). Development of a 3-plex droplet digital PCR for identification and absolute quantification of Salmonella and its two important serovars in various food samples. Food Control.

[40] Coudray-Meunier C., Fraisse A., Martin-Latil S., Guillier L., Delannoy S., Fach P., Perelle S. (2015). A comparative study of digital RT-PCR and RT-qPCR for quantification of Hepatitis A virus and Norovirus in lettuce and water samples. Int. J. Food Microbiol..

[41] Lei S., Chen S., Zhong Q. (2021). Digital PCR for accurate quantification of pathogens: Principles, applications, challenges and future prospects. Int. J. Biol. Macromol..

[42] Gansen A., Herrick A.M., Dimov I.K., Lee L.P., Chiu D.T. (2012). Digital LAMP in a sample self-digitization (SD) chip. Lab Chip.

[43] Lobato I.M., O’Sullivan C.K. (2018). Recombinase polymerase amplification: Basics, applications and recent advances. Trend. Anal. Chem..

[44] Xu X., Su Y., Zhang Y., Wang X., Tian H., Ma X., Chu H., Xu W. (2021). Novel rolling circle amplification biosensors for food-borne microorganism detection. Trend. Anal. Chem..

[45] Ma Y.-D., Chang W.-H., Luo K., Wang C.-H., Liu S.-Y., Yen W.-H., Lee G.-B. (2018). Digital quantification of DNA via isothermal amplification on a self-driven microfluidic chip featuring hydrophilic film-coated polydimethylsiloxane. Biosens. Bioelectron..

[46] Wu X., Tay J.K., Goh C.K., Chan C., Lee Y.H., Springs S.L., Wang D.Y., Loh K.S., Lu T.K., Yu H. (2021). Digital CRISPR-based method for the rapid detection and absolute quantification of nucleic acids. Biomaterials.

[47] Yin W., Zhuang J., Li J., Xia L., Hu K., Yin J., Mu Y. (2023). Digital Recombinase Polymerase Amplification, Digital Loop-Mediated Isothermal Amplification, and Digital CRISPR-Cas Assisted Assay: Current Status, Challenges, and Perspectives. Small.

[48] Chen L., Ding J., Yuan H., Chen C., Li Z. (2022). Deep-dLAMP: Deep Learning-Enabled Polydisperse Emulsion-Based Digital Loop-Mediated Isothermal Amplification. Adv. Sci..

[49] Yi C., Luo Z., Lu Y., Belwal T., Pan X., Lin X. (2021). Nanoporous hydrogel for direct digital nucleic acid amplification in untreated complex matrices for single bacteria counting. Biosens. Bioelectron..

[50] Xie Y., Chen Z., Cai D., Huang D., Huang E., Yang X., Zhang T., Wen H., Wang Y., Zhao M. (2024). Rapid Detection of Uropathogens Using an Integrated Multiplex Digital Nucleic Acid Detection Assay Powered by a Digital-to-Droplet Microfluidic Device. Anal. Chem..

[51] Moehling T.J., Choi G., Dugan L.C., Salit M., Meagher R.J. (2021). LAMP diagnostics at the point-of-care: Emerging trends and perspectives for the developer community. Expert Rev. Mol. Diagn..

[52] Kaminski M.M., Abudayyeh O.O., Gootenberg J.S., Zhang F., Collins J.J. (2021). CRISPR-based diagnostics. Nat. Biomed. Eng..

[53] Chen J.S., Ma E., Harrington L.B., Da Costa M., Tian X., Palefsky J.M., Doudna J.A. (2018). CRISPR-Cas12a target binding unleashes indiscriminate single-stranded DNase activity. Science.

[54] Wu H., Cao X., Meng Y., Richards D., Wu J., Ye Z., deMello A.J. (2022). DropCRISPR: A LAMP-Cas12a based digital method for ultrasensitive detection of nucleic acid. Biosens. Bioelectron..

[55] Park J. (2025). Rolling circle amplification as a molecular tool for spatially resolved signal amplification in single molecule counting assay. Biosensors.

[56] Gong F., Shan X., Tang Z., He Y., Zhou F., Ji X., He Z. (2025). A Compartmentalization-free digital immunoassay based on plasmonic-fluorescence nanoparticles. Anal. Chem..

[57] Ali M.M., Li F., Zhang Z., Zhang K., Kang D.-K., Ankrum J.A., Le X.C., Zhao W. (2014). Rolling circle amplification: A versatile tool for chemical biology, materials science and medicine. Chem. Soc. Rev..

[58] Deng R., Dong Y., Xia X., Dai Y., Zhang K., He Q., Zeng W.-c., Ren X., Li J. (2018). Recognition-enhanced metastably shielded aptamer for digital quantification of small molecules. Anal. Chem..

[59] Deng R., Shi Y., Zhang Y., Zhang X., Deng S., Xia X. (2024). Precise, sensitive detection of viable foodborne pathogenic bacteria with a 6-order dynamic range via digital rolling circle amplification. ACS Sens..

[60] Rolando J.C., Jue E., Schoepp N.G., Ismagilov R.F. (2019). Real-time, digital LAMP with commercial microfluidic chips reveals the interplay of efficiency, speed, and background amplification as a function of reaction temperature and time. Anal. Chem..

[61] Yue X., Fang X., Sun T., Yi J., Kuang X., Guo Q., Wang Y., Gu H., Xu H. (2022). Breaking through the Poisson Distribution: A compact high-efficiency droplet microfluidic system for single-bead encapsulation and digital immunoassay detection. Biosens. Bioelectron..

[62] Fozouni P., Son S., de León Derby M.D., Knott G.J., Gray C.N., D’Ambrosio M.V., Zhao C., Switz N.A., Kumar G.R., Stephens S.I. (2021). Amplification-free detection of SARS-CoV-2 with CRISPR-Cas13a and mobile phone microscopy. Cell.

[63] Liu T.Y., Knott G.J., Smock D.C.J., Desmarais J.J., Son S., Bhuiya A., Jakhanwal S., Prywes N., Agrawal S., Díaz de León Derby M. (2021). Accelerated RNA detection using tandem CRISPR nucleases. Nat. Chem. Biol..

[64] Wang M., Liu Z., Liu C., He W., Qin D., You M. (2024). DNAzyme-based ultrasensitive immunoassay: Recent advances and emerging trends. Biosens. Bioelectron..

[65] Wang Z., Shao Y., Zhu Z., Wang J., Gao X., Xie J., Wang Y., Wu Q., Shen Y., Ding Y. (2023). Novel gold nanozyme regulation strategies facilitate analytes detection. Coord. Chem. Rev..

[66] Wang Z., Cheng X., Ma A., Jiang F., Chen Y. (2025). Multiplexed food-borne pathogen detection using an argonaute-mediated digital sensor based on a magnetic-bead-assisted imaging transcoding system. Nat. Food.

[67] Wang Z., Ma A., Chen Y. (2024). An Amplification-Free Digital Assay Based on Primer Exchange Reaction-Mediated Botryoidal-Like Fluorescent Polystyrene Dots to Detect Multiple Pathogenic Bacteria. ACS Nano.

[68] Zhou Y., Zhao J., Chen R., Lu P., Zhao W., Ma R., Xiao T., Dong Y., Zheng W., Huang X. (2024). A portable deep-learning-assisted digital single-particle counting biosensing platform for amplification-free nucleic acid detection using a lens-free holography microscope. Nano Today.

[69] Romphosri S., Pissuwan D., Wattanavichean N., Buabthong P., Waritanant T. (2024). Rapid alignment-free bacteria identification via optical scattering with LEDs and YOLOv8. Sci. Rep..

[70] Terven J., Córdova-Esparza D.-M., Romero-González J.-A. (2023). A Comprehensive Review of YOLO Architectures in Computer Vision: From YOLOv1 to YOLOv8 and YOLO-NAS. Mach. Learn. Knowl. Extr..

[71] Quan H., Wang S., Xi X., Zhang Y., Ding Y., Li Y., Lin J., Liu Y. (2024). Deep learning enhanced multiplex detection of viable foodborne pathogens in digital microfluidic chip. Biosens. Bioelectron..

[72] Rissin D.M., Kan C.W., Campbell T.G., Howes S.C., Fournier D.R., Song L., Piech T., Patel P.P., Chang L., Rivnak A.J. (2010). Single-molecule enzyme-linked immunosorbent assay detects serum proteins at subfemtomolar concentrations. Nat. Biotechnol..

[73] Li Z., Hua L., Xie L., Wang D., Jiang X. (2023). Automated microfluidic nucleic acid detection platform-integrated RPA-T7-Cas13a for pathogen diagnosis. Anal. Chem..

[74] Deng R., Tang L., Tian Q., Wang Y., Lin L., Li J. (2014). Toehold-initiated rolling circle amplification for visualizing individual microRNAs in situ in single cells. Angew. Chem. Int. Ed..

[75] Cui F., Yue Y., Zhang Y., Zhang Z., Zhou H.S. (2020). Advancing biosensors with machine learning. ACS Sens..

[76] Falk T., Mai D., Bensch R., Çiçek Ö., Abdulkadir A., Marrakchi Y., Böhm A., Deubner J., Jäckel Z., Seiwald K. (2019). U-Net: Deep learning for cell counting, detection, and morphometry. Nat. Methods.

[77] Nehme E., Weiss L.E., Michaeli T., Shechtman Y. (2018). Deep-STORM: Super-resolution single-molecule microscopy by deep learning. Optica.

[78] Ramos L.T., Sappa A.D. (2025). A Decade of You Only Look Once (YOLO) for Object Detection: A Review. IEEE Access.

[79] Chuai G., Ma H., Yan J., Chen M., Hong N., Xue D., Zhou C., Zhu C., Chen K., Duan B. (2018). DeepCRISPR: Optimized CRISPR guide RNA design by deep learning. Genome Biol..

